# Impact of Listening to Indian Classical Music, or Rāgas, on the Electroencephalogram: A Meta-Analysis

**DOI:** 10.7759/cureus.49592

**Published:** 2023-11-28

**Authors:** Shrinit Babel, Suman Baral, Abhishek Srivastava

**Affiliations:** 1 Morsani College of Medicine, University of South Florida, Florida, USA; 2 Surgery, Lumbini Medical College and Teaching Hospital, Tansen, NPL; 3 Center for Physical Medicine and Rehabilitation, Kokilaben Dhirubhai Ambani Hospital and Medical Research Institute, Mumbai, IND

**Keywords:** electroencephalography (eeg), systematic review and meta analysis, neuromodulation, holistic medicine, integrative, raga, indian classical music, classical music

## Abstract

Ancient Indian classical music (ICM) has long been lauded and recognized for influencing emotional responses by influencing the human body's resonance. A meta-analysis of prospective case studies published in the last ten years on the effect of ancient Indian music rāgas on brain waves is investigated. This meta-analysis aimed to analyze published prospective studies investigating the effect of ancient Indian rāgas on EEG in healthy subjects.

The present study included prospective studies published since 2012. Studies were obtained by searching four databases, such as PsychINFO, PubMed, Google Scholar, and JSTOR, and searching related journals. Eligibility criteria included studies assessing the impact of listening to Indian classical music on the EEG. Primary outcomes were changes in the brain waves, frequency, and power and their relationship to activity-related arousal, attention, and mental tasks. The studies were analyzed according to the PRISMA guidelines.

There were a total of five included studies with 71 participants in the age range of 19-30, and the conditions for the test groups were generally similar except for varying types of rāgas used and time of day. Analysis of the data collected from 71 participants revealed that music interventions had statistically significant effects on increasing alpha activity and attention scores. Fractal analysis was sensitive enough to detect EEG brainwave changes while and after listening to the rāga musical intervention. Rāgas stimulate arousal in different areas of the brain, depending on the emotions they are designed to evoke. However, the synchronized studies together could not highlight a significant relationship between rāgas and EEG fractal dimension values.

Although the meta-analysis failed to reproduce the same results from the individual studies, potentially due to the small sample size and study variation, the meta-analysis opens doors to the potential of rāgas to elicit distinct emotions and serve as robust predictors of emotional response. Future studies can explore the therapeutic potential of various rāgas in the clinical setting, such as in the management of cognitive disorders and stress or in modulating heart rate variability and cognitive performance.

## Introduction and background

The bloom and progress of human civilization have always been accompanied and intertwined with music. It is widely acknowledged that music can influence the human body's resonance and trigger a unique emotional response [1]. Particularly, various properties of a musical piece can either align or conflict with the body’s own rhythms, thereby conferring a distinct effect [2]. Regardless, the effect of music on the human body is multifarious. Studies have found that in the brain alone, music can help release the ‘happy’ neurotransmitters, modulate emotional states and mood, and reduce the perception of pain [3,4]. Listening to music helps keep the neurons and synapses more active, as different parts of the brain are involved in processing music [4].

These aspects have improved the acceptance of music-based interventions and music therapy across specialties. In recent years, the focus has moved to explore different types of mindful music and their applications in the clinical setting.

One such form of mindful music that is gaining interest is Indian classical music (ICM), colloquially known as rāgas [5]. It is one of the oldest systems of music in the world, but it is still practiced today. Literally, the prefix ra refers to the ‘tune of life’, so rāga means the coloring of life [5]. Rāgas are considered to be a fluid form of music, known for how each musical note has guidelines yet is improvisational at the same time [5]. This aspect makes the soundscape unique from other forms of music commonly employed in music therapy interventions.

Particularly, both ICM and Western music employ the same notes but differ in their styles (Figure 4) [5]. Rāgas place an emphasis on melody and rhythm and are thus monophonic in nature [5]. Meanwhile, western music joins many notes together to form chords, making it polyphonic [5]. Rāgas also have significant droning, or instantaneous acoustic oscillations, which are produced from instruments like the Tanpura or Surpeti. This aspect is collectively known as the Tanpura drone [5].

Under the ICM genre, there are many families and forms of rāgas, whereby unique rāgas have been ascribed to a certain time of day or season [6]. For example, Rāg Bhairav is encouraged to be played in the morning [6]. All of these elements of ICM are designed uniquely for each rāga to evoke a particular emotion and modulate arousal at particular times of the day [6].

Past neurological studies have also identified that music therapies can be evaluated with electroencephalogram (EEG) studies to explore their impact on the brain system [7]. Each frequency band of the EEG rhythm relates to specific functions of the brain. For example, the frontal midline theta rhythm (Fm theta) often appears on the EEG during consecutive mental tasks [8]. The features of ICM have encouraged studies to investigate the impact of listening rāgas on the EEG. A randomized control trial investigating Rāga Desi Todi in healthy college students found a significant increase in alpha band activity and a decrease in anxiety and depression scores [9].

As the acceptance of music-based interventions and music therapy improves across specialties, the focus has also moved towards exploring different forms of mindful music and discovering their appropriate applications in the clinical setting. Given its different musical approach, ICM continues to garner attention, necessitating a review to assess the impact of listening to this genre of music on the EEG.

The aim of this study was to analyze published prospective case studies in the form of a meta-analysis to assess the impact of listening to rāgas on the EEG of healthy individuals.

## Review

Materials and methods

The review followed the Preferred Reporting Items for Systematic Reviews and Meta-Analyses (PRISMA) guidelines [10].

Search Strategy

The literature search was conducted using PsychINFO, PubMed, Google Scholar, and Web of Science electronic databases with criteria such as “EEG” AND “Indian Classical Music OR Rāgas” with dates from 2010 to January 2022. The search criteria were elaborated by employing the PICO tool to search for articles and determine their eligibility (Table 1).

**Table 1:** Population, intervention, comparison, and outcome (PICO) tool

PICO	Description	Search terms
Population	Healthy individuals (i.e., no chronic illnesses or neurological/audiological conditions)	Healthy Patients
Intervention	Indian classical music/Rāgas	Indian Classical Music OR Rāgas
Comparison	No music (negative control)	
Outcome	Primary outcome: changes in EEG and EEG fractal dimension analysis secondary outcome: moderating variables such as music preference	EEG or EEG fractal analysis

Study Eligibility Criteria

**Figure 1:**
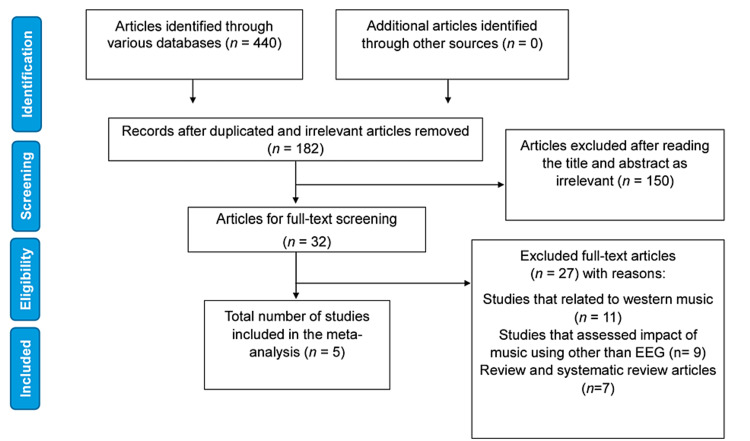
PRISMA flowchart

The inclusion criteria for the obtained studies were publicly published prospective studies that assessed the impact of Indian classical music/rāgas on the EEG in English (Figure 1). Studies that were excluded were studies that did not include ICM/rāgas as an intervention, did not assess the impact of listening to music using the EEG, or were themselves review articles or other gray literature studies. The studies were manually evaluated for their eligibility among the investigators collectively.

Data Extraction and Synthesis

Review Manager 5.3 (The Nordic Cochrane Centre, The Cochrane Collaboration, Copenhagen) was used to conduct the meta-analysis and organize the data, whereby an author independently performed the data extraction process after the studies were shortlisted. Study metadata such as the year of publication, sample size, gender, effect, and measures was extracted as an addition to the desired EEG-fractal dimension values for the meta-analysis. The EEG-fractal dimensions were evaluated for changes in brain waves, frequency, and power based on activity-related arousals. Study characteristics such as age group, listening time, type of rāga, and time of day were also tabulated to understand the degree of heterogeneity among the studies. Qualitative aspects of the results and additional measures of arousal (i.e., psychometrics) were also noted separately if they were employed in the study but not included in the meta-analysis.

Quality Assessment and Risk of Bias

The quality of the studies and their individual risk of biases were appraised using the Quality Assessment Tool for Quantitative Studies (QATQS) [11]. Two authors independently reviewed the selected studies using the QATQS tool and assigned a global rating to each study.

Results

Eligible Studies

The quality of the studies and their individual risk of biases were appraised using the QATQS [11]. Two authors independently reviewed the selected studies using the QATQS tool and assigned a global rating to each study.

Across the individual studies, the sample sizes ranged from two to 30, with a total of 71 participants across all five studies. Male participants were dominant in the review, with an average of 59% males across all five studies. Among the five studies included, there were three major musical interventions: Tanpura drone, Hindustani rāga, and Carnatic rāga. One study included two positive control groups of subjects listening to hard rock and jazz. The time-of-day participants received musical intervention, and other relevant baseline characteristics are tabulated (Table 2). Age distribution was measured differently across the studies (Table 2).

**Table 2:** Characteristics of the included studies

Study	Author	n	% Female	Age mean ± SD (min-max)	Intervention	Time of day	Global rating	EEG changes
1	Banerjee et al. [9]	21	20%	(20-25)	Tanpura Drone	Afternoon	Moderate	Significant changes in EEG-FD
2	Sanyal et al. [12]	2	0%	30.0	Hindustani Rāga	Afternoon	Weak	Increase in left frontal lobe arousal after Raga Chhayanat; increase in right frontal lobe arousal after Raga Darbari Kannada.
3	Maity et al. [13]	10	40%	(19-25)	Tanpura Drone	Afternoon	Weak	Increase in alpha and theta spectra width
4	Geethanjali et al. [14]	8	75%	20.0±0.4	Carnatic Rāga	Morning	Weak	Increase in beta power
5	Kumar et al. [15]	30	50%	24.8±7.4/27.6±6.4	Carnatic Rāga	Morning	Strong	Increased Alpha-wave frequency

Results of Individual Studies

Banerjee et al. [9] assessed the change in brain state when subjected to audio. The investigation employed fractal dimensions, which is a sensitive parameter capable of distinguishing brain states even with an acoustic signal of simple musical structure. The study by Sanyal et al. [12] analyzed the effect of Hindustani music on brain activity during normal relaxing conditions using EEG. The finding showed that arousal-based activities were enhanced while listening to Hindustani music of contrasting emotions. Maity et al. [13] study the different levels of neural activation in the human alpha and theta brain rhythms under the effect of simple acoustical stimuli using different EEG feature classification techniques such as wavelet transform (WT) and multifractal detrended fluctuation analysis (MFDFA). It was found that in all the frontal electrodes, alpha as well as theta complexity increases, as is evident from the increase in multifractal spectral width.

Geethanjali et al. [14] also assessed the effect of music on brain function rate based on the experimenter’s choice of music (jazz, carnatic, and hard rock) with or without mental workload. The results of the study showed that jazz and carnatic music significantly improve brain function as compared to hard rock during mental tasks. Kumar et al. [15] assessed the effect of Indian classical music on brain activity using EEG. This study found that there is an effect of music on alpha waves.

Synthesis

Five studies report the effect of ancient Indian rāgas using EEG before and after using a random effect model [9,12-15].

**Figure 2:**
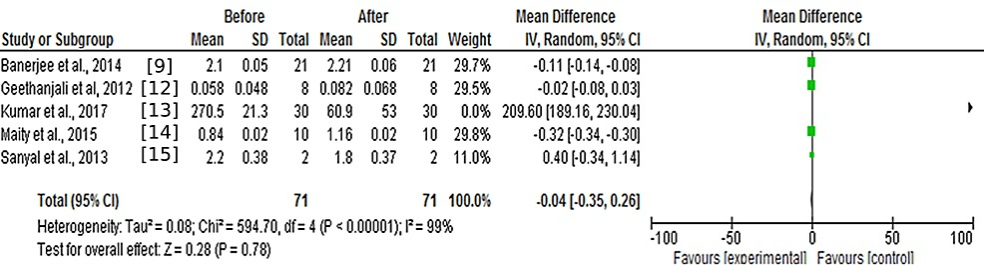
Random effect model on the effect of ragas on EEG fractal dimensions

**Figure 3:**
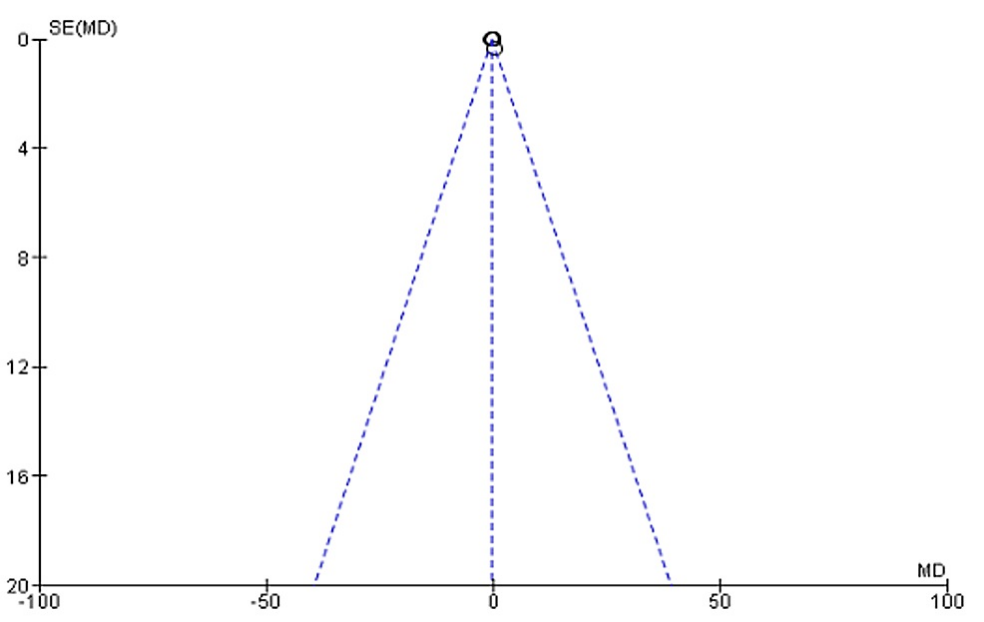
Funnel plot on the effect of ragas on EEG fractal dimensions

**Figure 4:**
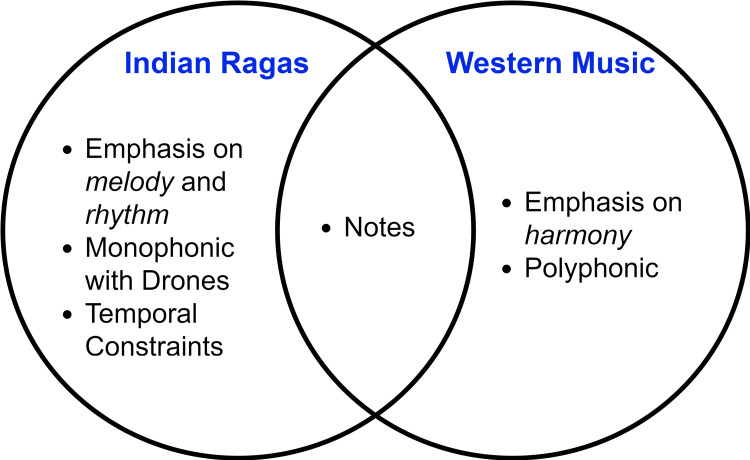
Comparison between Indian ragas and Western music

The meta-analysis indicated that, contrary to the findings of individual studies, there is no statistically significant difference in fractal dimensions observed before and after EEG. Test for overall effect: Z = 0.28 (MD = −0.04, CI: −0.35 to 0.26 (p = 0.78>0.05) (Figure 2). The funnel plot (Figure 3) suggests that most of the studies chosen had sufficient statistical power for analysis considering the context of what is being investigated.

Discussion

The individual studies were promising in demonstrating a relationship between the music intervention and EEG alpha activity along with attention scores. Fractal analysis was sensitive enough to detect EEG brainwave changes during and after listening to the rāga music intervention. However, the meta-analysis was inconclusive, unable to generate a statistically significant relationship between the music intervention and EEG brainwave changes. The discrepancy can be attributed to the fewer studies and varying study designs employed in each experiment. For example, the duration and time of day subjects received the rāga music intervention differed in every study. Preference or familiarity with a certain form of music could also be a factor in such EEG studies. From the studies, it was unclear whether the participants preferred rāgas or not.

Therefore, future studies can evaluate the impact of other variables, such as listening duration, participant preference, type of rāga, time of day, and specific physical and psychological participant covariates, to better delineate the potential of rāgas. Such variables, especially the time of day, can have significant effects. For example, music with an upbeat tempo can have different effects in the morning hours compared to the evening hours [16]. Participant preference is also a significant covariate and is often considered when developing guidelines for music-based interventions [17]. This can be coupled with randomized-controlled trials using particular rāgas lauded for their therapeutic properties as a music intervention in various contexts. The individual studies also did not mention the state of wakefulness among subjects or discuss in detail frontal rhythmic theta waves in adolescent subjects, both factors that are relevant to understanding the impact of the musical interventions.

One particular application of raga music therapy can be potential in comatose patients. The bioelectrical nature of the brain in comatose patients can be characterized by low amounts of high-frequency waves [18]. In fact, the EEG in comatose patients has prognostic significance: a more suppressed EEG is typically associated with a poorer prognosis, while the return of high-frequency waves may indicate higher chances for recovery [19].

As rāgas were found by some studies to increase the levels of alpha and beta waves [20], a study can evaluate the impact of listening to rāgas on the EEG of comatose or traumatic brain injury patients. Rāgas can also be evaluated in psychiatric patients, as one of the studies found that it increased attention scores among subjects, and another background study found a decrease in depression inventory scores. Likewise, music therapy has been previously found beneficial in schizophrenia patients, so a study can evaluate the benefits of listening to rāgas in such contexts [21,22].

Notably, while our meta-analysis specifically focused on the effects of rāgas, there are several similar studies analyzing brainwave changes upon listening to Nasyids (Nasheeds). Nasyids, akin to rāgas, place heavy emphasis on vocal expression but are not accompanied by musical instruments. Particularly interesting are two preliminary studies that paralleled the study by Geethanjali et al., comparing Nasyids to rock music [23] and Western classical music [24]. Both studies demonstrated a more pronounced increase in alpha band activity when listening to Nasyids as compared to rock music or Western classical music [23,24]. An additional study not only found comparable impacts on alpha band activity but also revealed that Nasyids have a mild moderating effect on diastolic blood pressure [25]. These findings can be coupled with our meta-analysis as another avenue of research. Future studies can explore advanced data science techniques, such as machine learning clustering, to identify the distinctive features of specific classes of mindful music and their correlation with brainwave changes [26]. This approach may provide a more nuanced understanding of the intricacies between sound characteristics and neurophysiological responses.

Rāgas employ unique pitch, time, and dynamics, along with their own spatial and temporal characteristics. This meta-analysis opens doors to the potential of rāgas to elicit distinct emotions and serve as robust predictors of emotional response. Future studies can explore the therapeutic potential of various rāgas in the clinical setting.

## Conclusions

In conclusion, the meta-analysis was inconclusive and contradicted the results of the individual studies. The individual studies found that South Asian ragas increase alpha activity and improve attention scores. The likely discrepancy could be attributed to the limited number of investigations involving this topic as well as slight variations across the study designs that could not have been statistically classified. One aspect that must be acknowledged is that there is a limited availability of studies on this topic. Regardless, this systematic review and meta-analysis opens doors to further investigation in the area to better understand the therapeutic potential of integrating various genres of music in the clinical setting.
